# Endosialin promotes vascular maturation by inhibiting Cyr61 expression in melanoma metastasis

**DOI:** 10.3389/fonc.2025.1528288

**Published:** 2025-07-25

**Authors:** Tong Lu, Hongtao Song, Zhite Zhao, Xinglin He, Chao Xu, Shaojie Liu, Weijun Qin, Bo Yang, Lijun Yang

**Affiliations:** Department of Urology, Xijing Hospital, Air Force Medical University, Xi’an, China

**Keywords:** Endosialin/CD248/TEM-1, Cyr61/CCN1, melanoma, angiogenesis, tumor metastasis

## Abstract

**Background:**

Extensive tumor cell metastasis is associated with poor patient prognosis. Tumor endothelial cells demonstrate distinct proangiogenic phenotypes compared to normal endothelial cells, partially mediated by pericyte-derived secreted factors. Endosialin, a pericyte biomarker implicated in vascular maturation and metastatic progression, remains mechanistically undefined in this context.

**Methods:**

B16F10 melanoma cells were injected via caudal vein into Endosialin knockout (EN^KO^) and wildtype mice. Lung metastases were quantified through hematoxylin-eosin (HE) staining. Vascular architecture was analyzed using Evans blue perfusion and CD31 immunofluorescence. Molecular mechanisms were investigated through western blotting, qPCR, proliferation assays, and *in vitro* lumen formation models.

**Results:**

Bioinformatics analysis revealed Endosialin overexpression correlates with enhanced angiogenesis and poor clinical outcomes. Endosialin deficiency significantly reduced pulmonary metastasis burden. Vascular profiling showed EN^KO^ mice exhibited increased small-diameter vessels (<50 μm) and reduced mature vessels (≥50 μm). Mechanistically, Endosialin regulates vascular maturation through Erk1/2-mediated suppression of Cyr61 in pericytes

**Conclusion:**

Endosialin facilitates melanoma metastasis by promoting vascular maturation via Erk1/2-Cyr61 signaling axis in pericytes.

## 1 Introduction

The tumor microenvironment (TME) represents a dynamic ecosystem comprising endothelial cells, fibroblasts, and immunosuppressive populations that collectively drive tumor progression. Within this milieu, tumor endothelial cells (TECs) emerge as key functional components, exhibiting unique angiogenic properties distinct from normal endothelium (1).

TECs can promote tumor metastasis in different ways (2). Abnormal morphology of tumor vasculature leads to tumor cell intravasation during tumor metastasis (3, 4). Besides, TECs isolated from highly metastatic tumors exhibit more significant stem cell-like phenotypes compared with TECs from low metastatic tumor (5). However, the molecular mechanism of inducing angiogenesis and promoting vascular maturation in metastases is still unclear.

Pericytes, the mural cells enveloping microvessels, participate in all phases of vascular remodeling - from developmental sprouting to pathological angiogenesis (6, 7). Their regulatory functions are mediated through direct cell-cell interactions and secretion of paracrine factors (8), with emerging evidence implicating pericyte-derived signals in pro-metastatic vascular normalization (9). Notably, therapeutic strategies targeting pericyte-tumor crosstalk have demonstrated remarkable preclinical efficacy (10, 11), underscoring their clinical relevance.

Endosialin (CD248/TEM-1), a transmembrane glycoprotein overexpressed on tumor-associated pericytes, has been paradoxically linked to both pro-fibrotic progression and vascular destabilization (12, 13). Preclinical models show Endosialin knockdown reduces metastatic burden while unexpectedly increasing microvascular density (12, 14–16), suggesting its unresolved role in vascular maturation.

Our mechanistic investigation reveals Endosialin deficiency attenuates melanoma metastasis and induces vascular immaturity (<50 μm diameter). We further identify a novel Erk1/2-Cyr61 regulatory axis through which Endosialin modulates pericyte-mediated vascular maturation, resolving previous mechanistic ambiguities.

## 2 Methods

### 2.1 Culture of cell lines

Human retinal pericytes cell line (HRMVP) and Human Umbilical Vein Endothelial Cell (HUVEC) were purchased from aoyinbio Co., Ltd (Shanghai, China). Cells were maintained in Dulbecco’s modified Eagle’s medium (DMEM) medium supplemented with 10% fetal bovine serum (FBS) (#A3161002C, Gibco) and 1% penicillin–streptomycin (#15070063, Gibco). ERK1/2 inhibitors (#HY-10256, MCE) were purchased. The concentration of ERK1/2 inhibitors (SB203580) was used by 10 μM *in vitro*.

### 2.2 Western blot and real time-quantitative PCR

Every protein sample was loaded on 8% SDS- PAGE and then transferred into a PVDF membrane (ThermoFisher Scientific). And then membrane incubated with primary antibodies overnight at 4°C: anti-EN (#ab48185, Abcam), anti-Cyr61 (#ab230947, Abcam), anti-GAPDH (#10494-1-AP, Proteintech), anti-p-MAPK (#4370T, CST), and anti-MAPK (#4695T, CST). Cells were harvested and total RNA was isolated with kit (#R6834010000J28U100, omegabio). Reverse transcription was conducted using PrimeScript™ RT Master Mix (TaKaRa, Japan). Then quantitative PCR was performed using an SYRB GreenII kit (#DRR041A; TaKaRa, Japan). The following primers were used: human-*Endosialin*-forward (F): 5′- CTCCACACATTCGTGTTCGC- 3′ and reverse (R): 5′- CTGCTACGCTCTCTTCCCAC- 3′; human-*CYR61*-F: 5′- CAGGTGGGTGGGATGTGAA- 3′ and R: 5′- GGGAAACGCTGCTTCATTGG- 3′; and human-*GAPDH*- F: 5′- GCAACTAGGATGGTGTGGCT- 3′ and R: 5′- TCCCATTCCCCAGCTCTCATA- 3′.

### 2.3 Animal experiments

All animal experiments of this study were approved by the Guidelines for the Care and Use of Laboratory Animals of Air Force Medical University. C57BL/6-*Endosialinem1Smoc* mice (systemic knockout) were purchased from Shanghai model organism company(#NM-KO-200094) and maintained in a 12h light/12h dark cycle with free access to food and water. And we choose littermate wild-type mice as control group. Each mouse was male and whether gender influences tumor promotion by Endosialin is unknown. Each mouse was inoculated with 2×10^6^ tumor cells (B16F10) in abdomen waiting for lung metastasis. The mice were sacrificed after 14 days.

### 2.4 Immunofluorescence staining

The primary antibodies used for IF and IHC staining were anti-CD31 (#77699, CST), anti-Endosialin (#ab204914, Abcam), anti-Cyr61(#1111, ABclonal) and anti-NG2(#A24955, ABclonal).

### 2.5 Cell proliferation assay and tube formation assay

Matrigel (BD Biosciences) was added to a 48-well plate (150 μL/well). HUVEC cells were required to be serum starved in advance for 6 h and collected after 0.25% trypsin (Invitrogen) digestion. Fifty thousand HUVECs were plated onto Matrigel in 300 μL of PBS/2% FBS per well at the time of plating. The cells were incubated at 37°C with humidified 95% air/5% CO2 for 6 h. The tubes/networks were imaged using Metamorph image analysis software (Molecular Devices, Sunnyvale, CA). Endosialin of HRNVP was knocked-down by si-RNA (GenePharma Co., Shanghai, China) before we collected culture supernatants. Cell proliferation assay was performed by the same way and cell counting kit-8 was purchased from mishubio, Xian, China. Cyr61 combination protein was purchased from Beyotime Biotechnology and the effective concentration was 3μg/mL.

### 2.6 Evans blue dye extravasation assay

Mice were injected with 2mg/kg lipopolysaccharide to induce systemic blood vessel leakage. 24h later, 1% (weight/volume) Evans blue dye was injected into the peritoneum. Two hours later, tumors of the mice were collected, weighted and treated with formamide (Sigma Aldrich) for 48h at 50°C. Optical density of Evans blue dye absorbance released from tissue was measured at 600nM using a spectrophotometer.

### 2.7 Bioinformatics analysis

Analysis of single gene differences in TCGA database was prepared for GSEA using DESeq. 2 package (version 1.26.0). In addition, GO and KEGG pathway enrichment analyses were predicted by the DAVID online database (https://david.ncifcrf.gov/). Single-cell data are derived from GSE72056 and GSE174401. Analysis of scRNA-seq datasets was performed primarily using the Seurat package (v4.0.5) in R (v4.1.0) and code was same with our previous article (17).

### 2.8 Statistics

Results were depicted as mean ± SEM. Comparisons were analyzed by two-tailed Student t test or one-way ANOVA followed by Dunnett’s *post hoc* test. Differences were considered significant at P<0.05.

## 3 Results

### 3.1 Endosialin is associated with angiogenesis in melanoma metastases

**Figure 1:**
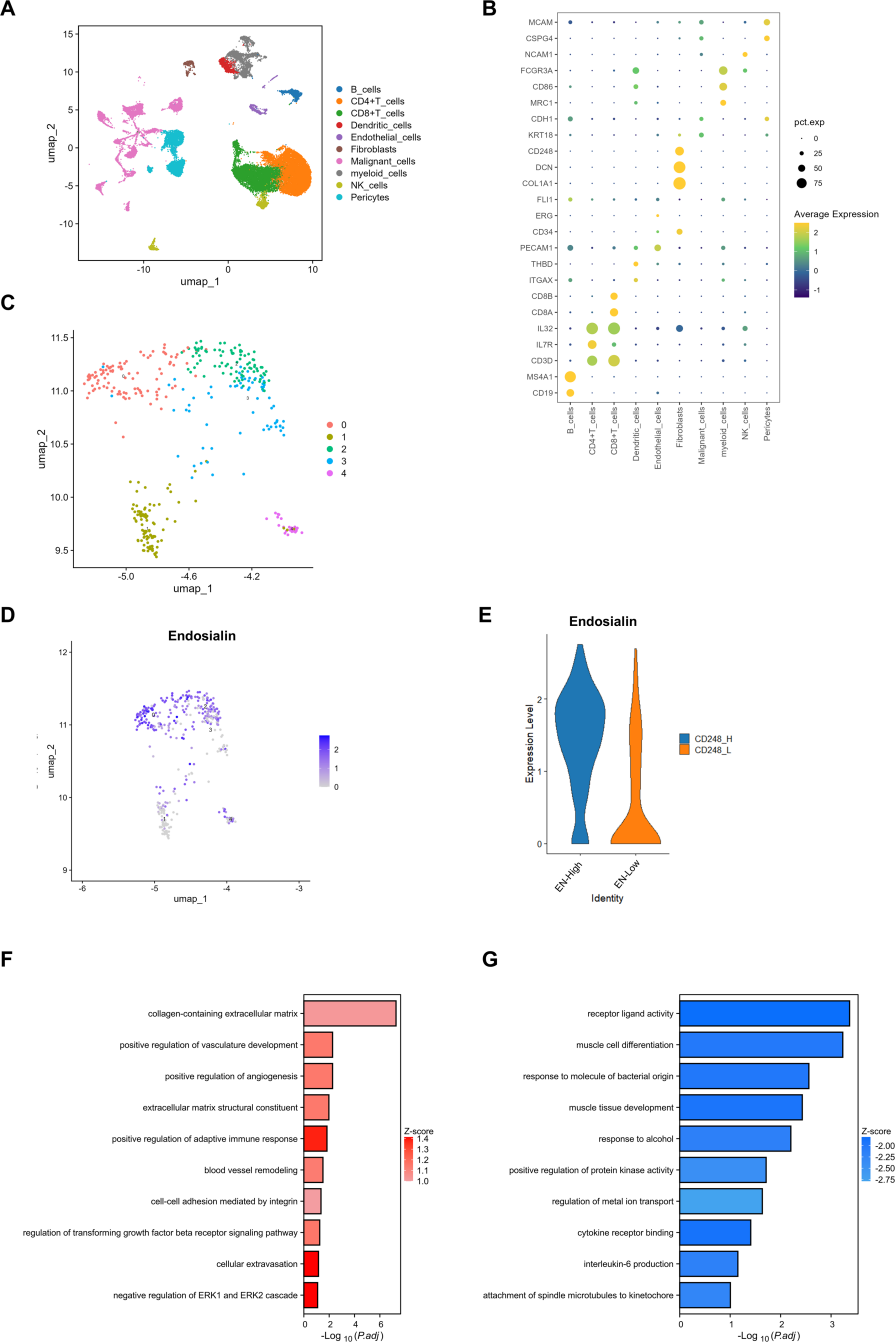
Endosialin is associated with angiogenesis in melanoma metastases. **(A)** UMAP visualization of the cell populations in tumor metastases from two scRNA-seq datasets which include 61patient specimens. **(B)** Bubble plot to show the DEGs in each cluster. **(C)** UMAP visualization of fibroblast clusters. Different fibroblast clusters are color-coded. **(D)** UMAP visualization of cell atlas to reveal the expression of Endosialin in each fibroblast subcluster. **(E)** Violin plots to show the expression levels of ENDOSIALIN in EN^high^ group and EN^low^ group. **(F)** GO enrichment analysis to show up-regulated gene in EN^high^ group. **(G)** GO enrichment analysis to show up-regulated gene in EN^low^ group.

Firstly, we integrated two single-cell sequencing datasets. These samples are all from metastatic lesions of melanoma, including lung and brain metastases (**Supplementary Figure S1A**). We identified 10 cell clusters and their characteristic genes (**Figures 1A, B**). During the tumor metastasis, tumor cells have a strong interaction with the microenvironment of the colonization site. Studies have shown that pericytes have a strong role in promoting tumor metastasis, so we analyzed Pericytes in the tumor microenvironment. We found that in metastatic lesions, Pericytes were divided into five subpopulations (**Figure 1C**). Our previous study found that Endosialin was a specific marker of Pericytes, which was also found in melanoma metastasis (**Supplementary Figure S1B**). We further divided the Pericytes into EN-high expression group and EN-low expression group according to the expression degree of Endosialin (**Figures 1D, E**). GO enrichment analysis showed that EN-high Pericytes were associated with extracellular matrix structural constituent and blood vessel remodeling (**Figure 1F**). EN-low Pericytes are associated with cytokine receptor binding and protein kinases activity (**Figure 1G**).

### 3.2 Patients with high expression of Endosialin have a worse prognosis and Endosialin is involved in angiogenesis in melanoma

**Figure 2:**
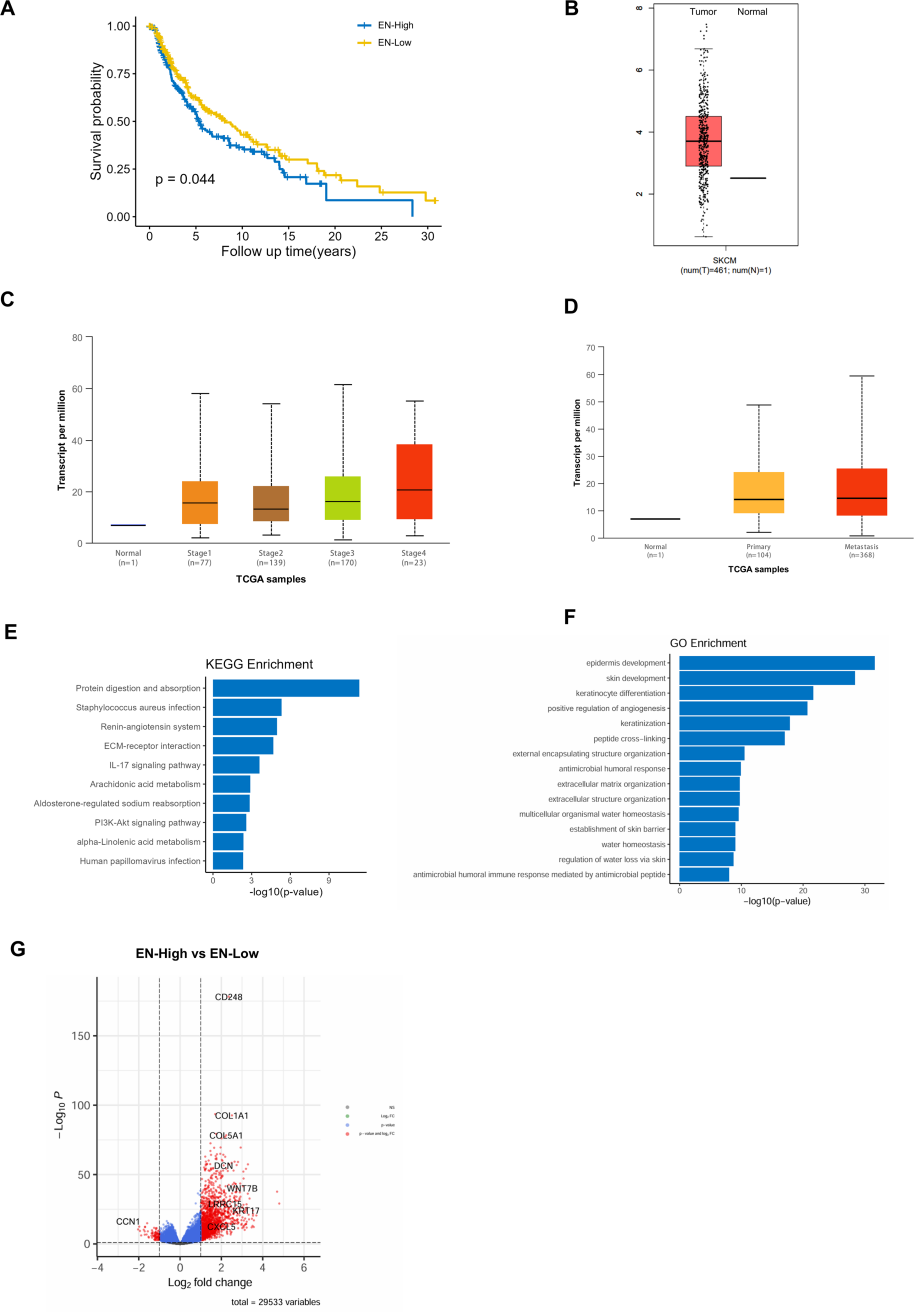
Patients with high expression of Endosialin have a worse prognosis and Endosialin is involved in angiogenesis in melanoma. **(A)** High expression of Endosialin represents the worse prognostic. **(B)** The expression of Endosialin upregulates in melanoma compared with normal tissues. **(C)** The expression of Endosialin is higher in stage 4 than the other stages. **(D)** The expression of Endosialin is higher in metastases than primary tumor. **(E)** KEGG enrichment analysis of EN^high^ patient group. **(F)** GO enrichment analysis of EN^high^ patient group. **(G)** Up regulation gene in EN^high^ group and EN^low^ group.

Using the TCGA database, we found that high expression of Endosialin was associated with worse prognosis in melanoma patients (**Figure 2A**). It was further found that Endosialin expression was higher in tumor tissues compared with normal tissues (**Figure 2B**). Next, we compared patients at different periods of disease progression and found that ENDOSIALIN expression was higher in patients at advanced clinical stages compared with patients at earlier stages (**Figure 2C**). In addition, the expression of Endosialin was higher in the metastases compared with the primary tumor (**Figure 2D**). These results suggested that Endosialin may be a driven factor of tumor metastasis to promote tumor progression.

To further explore the functions of Endosialin, we divided melanoma patients into high expression group and low expression group. And then, we performed GO and KEEG enrichment analysis of the differential genes between two groups. The results showed that Endosialin was involved in extracellular matrix receptor interaction and positive regulation of angiogenesis (**Figures 2E, F**). Among the differential genes, the expression of CCN1, which was related to angiogenesis, is down-regulated when Endosialin was highly expressed (**Figure 2G**). These results revealed that Endosialin is involved in angiogenesis.

### 3.3 Endosialin promotes vascular maturation and tumor metastasis in melanoma

**Figure 3:**
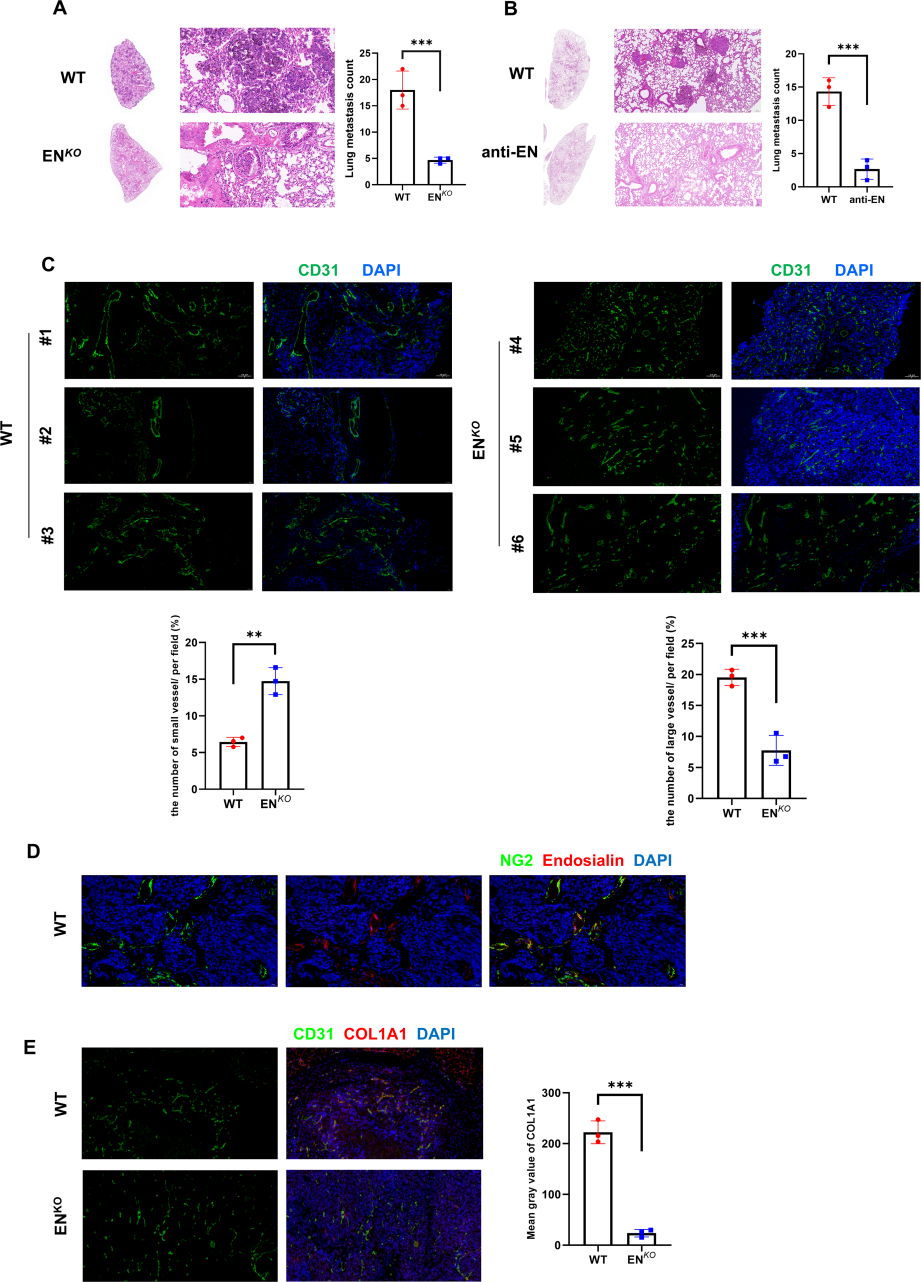
Endosialin promotes vascular maturation and tumor metastasis in melanoma. **(A)** Representative images of HE staining to show lung metastases in EN*^KO^* and WT mice. **(B)** Representative images of HE staining to show lung metastases in anti-EN and WT mice. **(C)** Immunofluorescence staining of CD31 in EN*^KO^* and WT mice. **(D)** Immunofluorescence staining of NG2 and Endosialin in WT mice to show co-localization. **(E)** Immunofluorescence staining of CD31 and COL1A1 in EN*^KO^* and WT mice. **means P<0.001, ***means P<0.0001.

To explore the function of Endosialin in metastasis, we inoculated melanoma cells (B16F10) into caudal vein of Endosialin knockout (EN*^KO^*) mice and wildtype (WT) mice. We found that EN*^KO^* mice had significantly fewer lung metastases compared with WT mice (**Figure 3A**). Similarly, we used the Endosialin blocking antibody after injection of tumor cells and found that the lung metastases in the anti-EN group were reduced (**Figure 3B**). The successful colonization of tumor cells in metastases depends on the rich vascular structure (2, 18). And Endosialin has been reported to promote vascular maturation (19). Therefore, we examined the vessel density and found that vessels of diameter <50μm increased and vessels of diameter ≥50μm decreased in EN*^KO^* mice (**Figure 3C**). To further detect the blood perfusion situation of lung metastasis, we injected Evans blue dye to tumor bearing mice, collected the metastasis 2h later and eluted the dye with formamide. We found that the blood perfusion of EN*^KO^* mice was relatively insufficient compared with WT mice (**Supplementary Figure S2A**). In melanoma, Endosialin is mainly expressed on pericytes (**Figure 3D**). We further examined the changes of all stromal cells in tumor tissues. We found that the number of stromal cells in EN*^KO^* tumor tissues decreased, which partly explained the changes in tumor microenvironment and vascular maturation disorders (**Figure 3E**). These results indicate that the loss of Endosialin leads to vascular maturation disorder, hypoperfusion of blood flow and, in turn, reduced tumor metastasis.

### 3.4 Endosialin regulates Cyr61 through Erk1/2 signaling pathway

**Figure 4:**
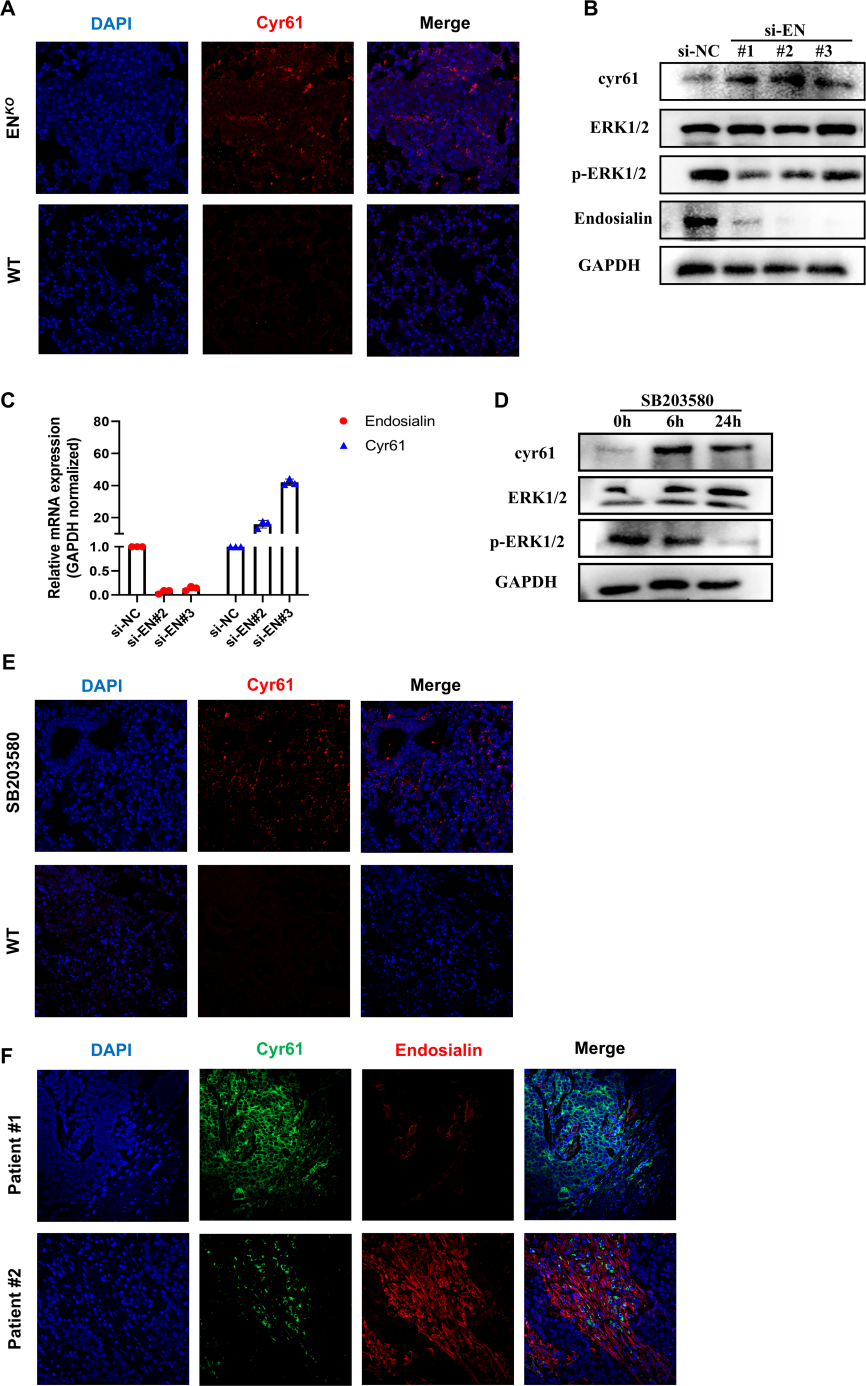
Endosialin regulates Cyr61 through Erk1/2 signaling pathway. **(A)** Immunofluorescence staining of Cyr61 in ENDOSIALIN*^KO^* and WT mice. **(B)** Western blot of knockdown Endosialin in HFL1. **(C)** RT-PCR of knockdown Endosialin. **(D)** Western blot of inhibiting phosphorylation of Erk1/2. **(E)** Immunofluorescence staining of Cyr61 in MAPK inhibitor group and WT mice. **(F)** Immunofluorescence staining of Cyr61 and Endosialin in patient tumor tissues.

Based on the changes of tumor vessels in mice and bioinformatics data, we examined whether Endosialin has a regulatory effect on Cyr61. It was reported that Cyr61 played a key role in angiogenesis (4, 20). In EN*^KO^* mice, the expression of Cyr61 increased (**Figure 4A**). Consistently, after knockdown Endosialin in HRMVP cell line, the phosphorylation of Erk1/2 was downregulated while the expression of Cyr61 was upregulated (**Figure 4B**). Quantitative PCR showed the same results (**Figure 4C**). Following the use of inhibitors of Erk1/2, downregulated phosphorylation of Erk1/2 and upregulated expression of Cyr61 were similarly found (**Figures 4D–E**). Besides, we collected two patients’ tissues. We found that there was a negative correlation between the expression of Endosialin and Cyr61 (**Figure 4F**). These results suggested that Endosialin regulated Cyr61 expression via the Erk1/2 signaling pathway.

### 3.5 Endosialin inhibits angiogenesis by downregulating Cyr61

**Figure 5:**
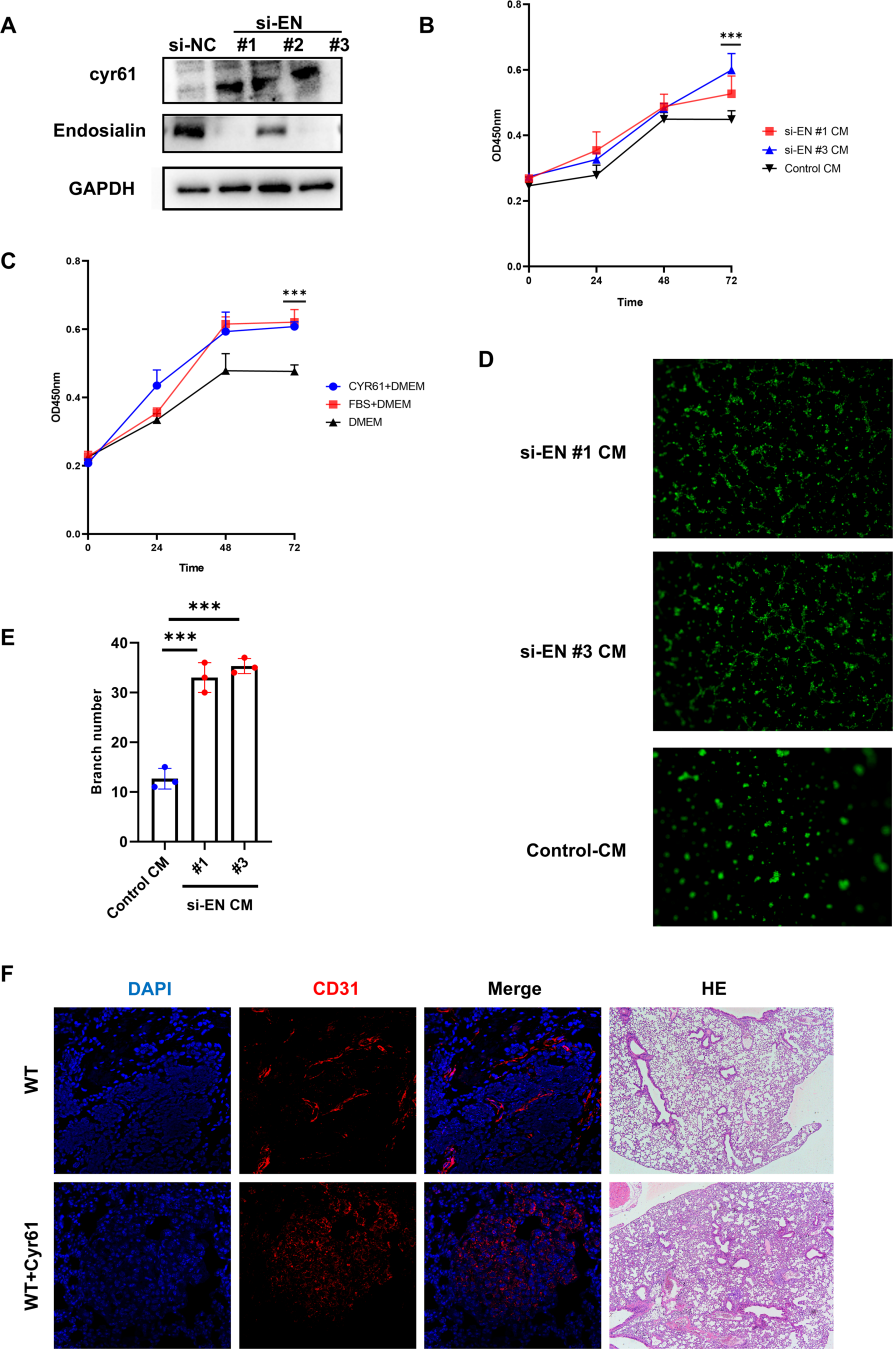
Endosialin inhibits angiogenesis by downregulating Cyr61. **(A)** Western blot of knockdown Endosialin in HRMVP. **(B)** Proliferation ability of HUVEC by CCK8 is performed in conditional medium. **(C)** Proliferation ability of HUVEC by CCK8 is performed in Cyr61 protein. **(D, E)** Tube formation ability of HUVEC is performed in conditional medium and related statistical charts. **(F)** Immunofluorescence staining of CD31 in Cyr61 recombinant protein group and WT mice. *** means P<0.0001.

We have known that the loss of Endosialin was able to increase small vessels *in vivo* and Endosialin can regulate the expression of Cyr61. In order to furtherly clarify the effects of Endosialin on endothelial cells, we knocked down the expression of Endosialin on HFL1 and collected the culture supernatant as conditioned medium (CM) (**Figure 5A**). We found that the CM of si-EN groups was able to promote the proliferation of endothelial cells (HUVEC) (**Figure 5B**). Since Endosialin was able to regulate expression of Cyr61, we examined the effects of Cyr61 on endothelial cells. We added the recombinant protein of Cyr61 into blank medium, which was able to promote the proliferation of HUVECs, achieving similar effects as those in the complete medium with 10% serum (**Figure 5C**). We also found that the CM of si-EN groups was able to promote tube forming ability of endothelial cells (**Figures 5D, E**). Besides, the use of Cyr61 recombinant protein in WT mice promoted tumor metastasis and small vessel formation (**Figure 5F**). These results suggested that Endosialin was able to suppress the proliferation of endothelial cells through inhibiting the expression of Cyr61.

## 4 Discussion

Cancer-related death is mainly attributed by tumor metastasis which seems to be incredible even though there has been emerged plenty of cancer therapies including surgical resection, chemotherapy, and radiotherapy (21). Therefore, understanding the mechanism of metastasis is very important. The metastatic process of tumor cells can be divided into 5 steps. Tumor cells acquire exercise capacity by epithelial mesenchymal transition (EMT) and invade into the surrounding ECM. And then, they penetrate blood vessels into the bloodstream. Circulating tumor cells (CTCs) manage to survive in the blood circulation and escape from anoikic resistance. When they reach distant organs through the circulation, they invade into the distant tissues. At last, tumor cells colonize and adapt to the new microenvironment and start proliferation (22).

EMT is the first step in the cascade process of tumor metastasis. EMT is a group of cell biological programs that endow tumor epithelial cells with the ability to degrade ECM (23). Tumor cells with EMT properties are required to move to the perivasculature and break through the vascular basement membrane between endothelial cells. Strong crosstalk with a variety of cells occurs in the stroma, including tumor derived pericytes (TDPs), macrophages, tumor endothelial cells (TECs) and other cells. These nonmalignant stromal cells exhibit an abnormal phenotype compared with normal tissues (24).

TDPs participate in the whole process of angiogenesis including sprouting and maturation of blood vessels. TDPs regulates maturation of vessels through autocrine, paracrine and direct cell-cell contact (8). TDPs are similarly able to promote tumor metastasis.

Endosialin is able to promote apoptosis of branched vessels and vessel maturation (12). What’s more, Endosialin expressed on the side of the basement membrane of pericytes is able to promote the process of tumor cell injection into blood vessels (19). Our study similarly confirms that Endosialin plays an important role in promoting melanoma to undergo lung metastasis. When Endosialin was deleted, there were more micro vessels and angiogenesis proteins concomitantly upregulated while metastasis of the tumor was reduced. Although our study still cannot reveal the mechanism about how Endosialin of TDPs is involved in tumor metastasis, we think it is related to abnormal vascular structure. This will be the direction of our subsequent studies. In our data, we found that CD248 inhibits the formation of small blood vessels and promotes large blood vessels, that is, the increase of mature blood vessels. These results are consistent with previous studies (12, 14). However, recent studies have shown that CD248 promotes angiogenesis in lung cancer (25). After carefully reading their research, we found that they only counted mature large blood vessels and ignored small blood vessels. Therefore, they draw the opposite conclusion (The following figure). In general, there is no substantial conflict between our research and previous studies. CD248 on pericytes inhibits the formation of small blood vessels and increases large blood vessels. And we reveal the mechanism behind this phenotype.

Abnormal vascular proliferation is one of the characteristics of tumors, and anti-vascular therapy remains to be a first-line treatment option for many tumors. In recent years, therapeutic regimens that promote vascular normalization have shown surprisingly anti-tumor effects in a variety of tumors (26). Vessel normalization usually refers to that the number of micro vessels is remarkably reduced and the coverage of pericytes is increased, so that the blood perfusion is significantly improved (27). The permeability of blood vessels is similarly reduced, which facilitate more drug delivering to the tumor (28).

The current study shows that high expression of Endosialin is associated with worse prognosis in patients and that antibody treatment strategies against Endosialin show robust antitumor responses (29–31). Therefore, although Endosialin seems to promote the maturation of tumor vessels, it remains to be clarified whether Endosialin brought about alterations in vascular structure indeed promote vessel normalization.

## Data Availability

The original contributions presented in the study are included in the article/**Supplementary Material**. Further inquiries can be directed to the corresponding authors.

## Supplementary material

Supplementary Figure 1 Data Integration and expression of Endosialin. **(A)** UMAP visualization of two datasets to show effective integration. **(B)** UMAP visualization of Endosialin expression.

Supplementary Figure 2 Evans Blue staining **(A)** Representative pictures and absorbance of Evans Blue dye after elution.
