# Supplementary figures and images for: Endosialin promotes vascular maturation by inhibiting Cyr61 expression in melanoma metastasis
Source: Front Oncol. 2025 Jul 25;15:1528288. doi: 10.3389/fonc.2025.1528288 (PMC12331472; doi:10.3389/fonc.2025.1528288)

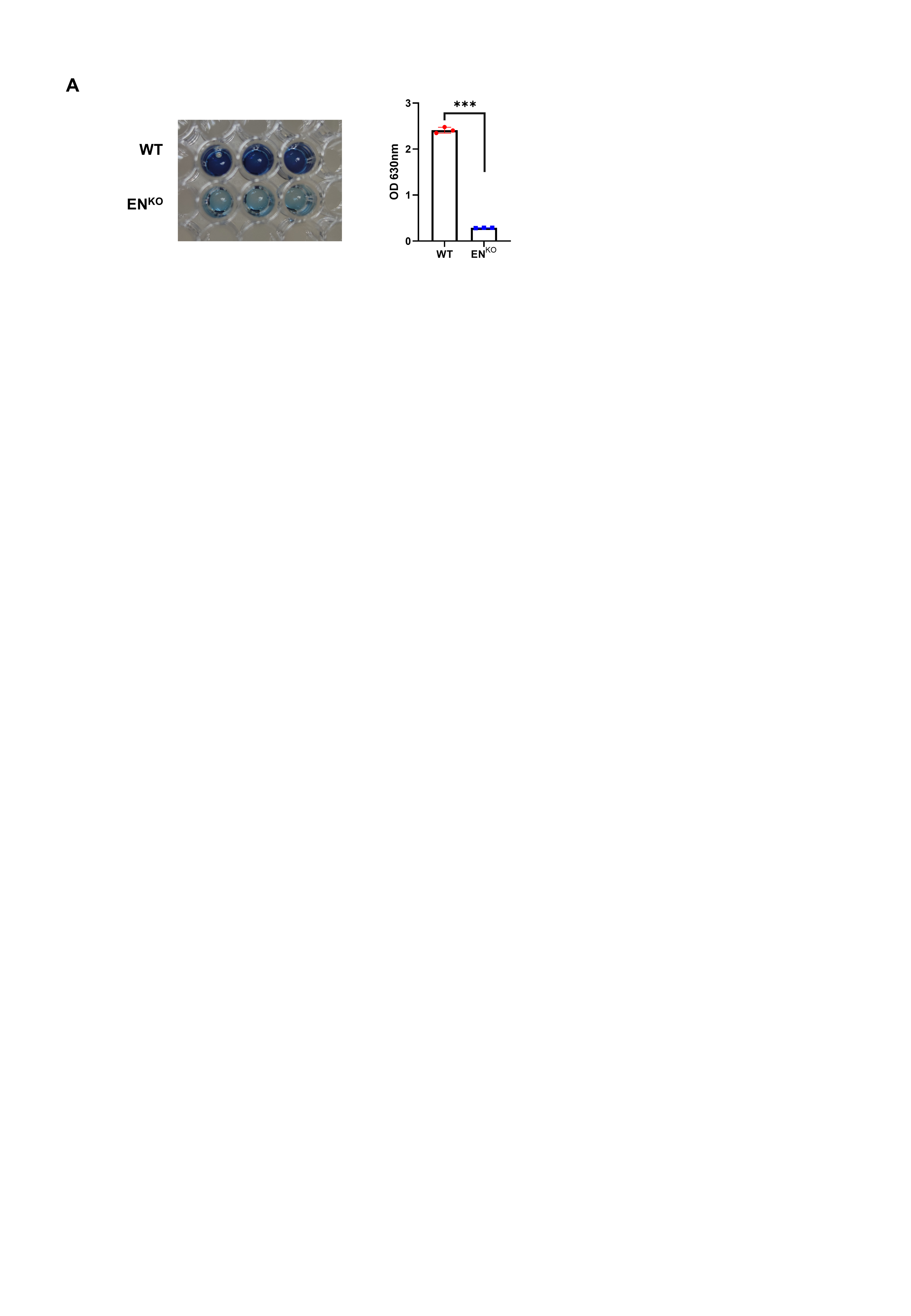

Supplement: Supplementary Figure 1 — Data Integration and expression of Endosialin. (A) UMAP visualization of two datasets to show effective integration. (B) UMAP visualization of Endosialin expression. [file Image1.tif]

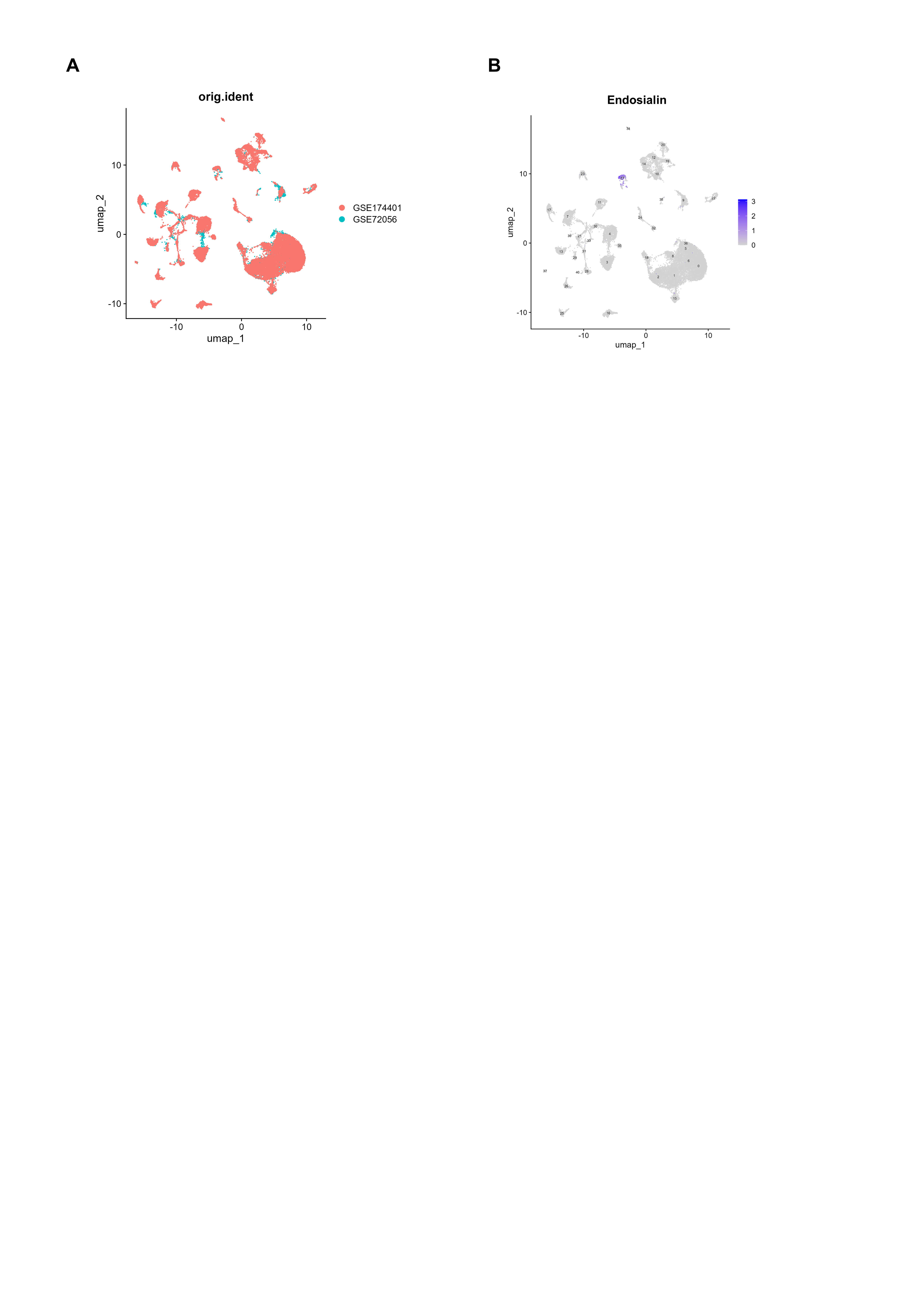

Supplement: Supplementary Figure 2 — Evans Blue staining (A) Representative pictures and absorbance of Evans Blue dye after elution. [file Image2.tif]
